# The Chromatin Remodelling Complex B-WICH Changes the Chromatin Structure and Recruits Histone Acetyl-Transferases to Active rRNA Genes

**DOI:** 10.1371/journal.pone.0019184

**Published:** 2011-04-29

**Authors:** Anna Vintermist, Stefanie Böhm, Fatemeh Sadeghifar, Emilie Louvet, Anethe Mansén, Pergiorgio Percipalle, Ann-Kristin Östlund Farrants

**Affiliations:** 1 Department of Cell Biology, The Wenner-Gren Institute, Stockholm University, Stockholm, Sweden; 2 Department of Cell and Molecular Biology, Karolinska Institutet, Stockholm, Sweden; Ludwig-Maximilians-Universität München, Germany

## Abstract

The chromatin remodelling complex B-WICH, which comprises the William syndrome transcription factor (WSTF), SNF2h, and nuclear myosin 1 (NM1), is involved in regulating rDNA transcription, and SiRNA silencing of WSTF leads to a reduced level of 45S pre-rRNA. The mechanism behind the action of B-WICH is unclear. Here, we show that the B-WICH complex affects the chromatin structure and that silencing of the WSTF protein results in a compaction of the chromatin structure over a 200 basepair region at the rRNA promoter. WSTF knock down does not show an effect on the binding of the rRNA-specific enhancer and chromatin protein UBF, which contributes to the chromatin structure at active genes. Instead, WSTF knock down results in a reduced level of acetylated H3-Ac, in particular H3K9-Ac, at the promoter and along the gene. The association of the histone acetyl-transferases PCAF, p300 and GCN5 with the promoter is reduced in WSTF knock down cells, whereas the association of the histone acetyl-transferase MOF is retained. A low level of H3-Ac was also found in growing cells, but here histone acetyl-transferases were present at the rDNA promoter. We propose that the B-WICH complex remodels the chromatin structure at actively transcribed rRNA genes, and this allows for the association of specific histone acetyl-transferases.

## Introduction

Transcription of ribosomal DNA (rDNA) constitutes the major transcriptional activity in eukaryotic cells, and occurs from large pre-ribosomal genes located in tandem repeats in the nucleolus. The nucleolus is also the location of processing of the 47/45S rRNA into three of the four rRNAs and the assembly of ribosomal subunits [1], [2]. Not all genes are active: approximately half are inactivated [3] by histone marks and methylated DNA in differentiated cells [1], [4]. The rRNA genes are transcribed by a specific transcription machinery employing RNA polymerase I (RNA pol I) with associating factors, such as UBF (Upstream binding factor) and SL1 (Selectivity factor 1); where UBF is required to bind at the enhancer region, and the SL1 complex (which contains TBP) at the promoter. The UBF binds not only at the promoter, but also in the transcribed region, and is involved in the formation of an open chromatin structure at actively transcribed genes [5]–[7]. In addition to UBF, chromatin remodelling complexes and histone-modifying protein complexes contribute to the chromatin structure at rDNA, in particular in the silencing of gene copies [1], [2], [8]. The chromatin remodelling complex NoRC, which consists of TIP5 and SNF2h, is the key regulator in the silencing of rRNA transcription in mammalian cells, where it is recruited by a non-structural RNA and TTF-1 [9], [10]. The silencing of the ribosomal genes follows the epigenetic changes that occur on RNA pol II genes and intergenic regions outside the nucleoli. After being recruited, the NoRC complex recruits DNA methyl-transferases (DMNTs), histone methyl-transferases and histone deacetylases (HDACs), resulting in methylated promoters with histone marks associated with silent genes. These include deacetylated histone H4, histone H3 methylated at lysine 9 (H3K9-me), histone H3 methylated at lysine 27 (H3K27-me), and histone H4 methylated at lysine 20 (H4K20-me). The recruitment of heterochromatin proteins also plays a role [11]–[13].

The active genes rely on the association of UBF to the promoter and coding region, either by interaction directly with DNA or with nucleosomes, creating a decondensation of the chromatin structure [7]. Even though the ribosomal transcription is high, it fluctuates with cellular state, being regulated by growth-factor signalling, nutrient state and stress [14]–[17]. Studies suggest that the regulation is not achieved by changing the ratio between silent and active genes, but rather by adjusting the transcription level of already active copies [18], [19]. The mechanism behind is less clear, and some studies have shown that changes in activity correlate with changes in the histone-acetylation levels at the promoter, while other studies have suggested a mechanism independent of nucleosomes and histone modifications [7]. Two ATP-dependent chromatin remodelling activities have been suggested to be involved in the activation of ribosomal genes: the B-WICH complex, consisting of a core of William syndrome transcription factor (WSTF), SNF2h and nuclear myosin 1 (NM1) [20], [21], and the CSB IP/150 complex, comprising Cockayne syndrome protein B (CSB), TFIIH and TIF 1B [22]. CSB plays dual roles in RNA pol I transcription: together with TFIIH it affects the elongation rate in an ATP-independent manner, and it remodels chromatin in an ATP-dependent manner [23]. The CSB, together with TFIIH and TIF1B, recruits the methyl-transferase G9a, resulting in H3K9-me2, which in turn recruits HP1γ, and these changes contribute to an activation of RNA pol I transcription [24]. The action of CSB suggests that histone modifications are also involved in the activation of ribosomal genes, although H3K9-me2 is not a clear activating histone modification in RNA pol II transcription [25]. The role of B-WICH in RNA pol I transcription is unknown.

The B-WICH complex is an extended form of WICH [26], and is involved in both RNA pol I and RNA pol III transcription [20], [21]. In addition to the three core proteins, WSTF, SNF2h, and nuclear myosin (NM1); the myb binding protein 1b, RNA helicase II/DXX21, and SAP155 all also associate via RNA species [21]. The subunit SNF2h is an ISWI ATPase, which slides nucleosomes in an ATP-dependent manner [27]. WSTF is a component of several complexes: two SNF2h complexes, B-WICH [21] and WICH [26], and one SWI/SNF type of chromatin remodelling complex, the WINAC complex, which is involved in vitamin D-mediated RNA pol II transcription [28]. The switch between WSTF associating to SNF2h and the SWI/SNF complex was recently described as being caused by phosphorylation of the WSTF protein by the MAP-kinase pathway [29]. WSTF is also a tyrosine kinase, phosphorylating H2Ax prior to DNA damage [30].

In this study, we have investigated the mechanism by which the B-WICH complex is involved in the transcription of the RNA pol I genes. We show that WSTF affects the chromatin structure over the ribosomal promoter and thereby influences the state of histone modifications. One effect is a reduction in the levels of H3-Ac, in particular H3K9-Ac, at the rRNA promoter found in WSTF knock down cells. Acetylation of histones is a modification that is associated with active transcription and open chromatin in RNA pol II transcription, and our results show that a similar mechanism is in operation at the rDNA locus. We show also that WSTF is required for the recruitment of certain histone acetyl-transferases (HATs), PCAF, p300, and GCN5, and we suggest that B-WICH functions by preferentially facilitating the access of specific factors to the DNA, where they contribute to the active state of rRNA.

## Results

### WSTF, SNF2h and NM1 associate with active rDNA

**Figure 1:**
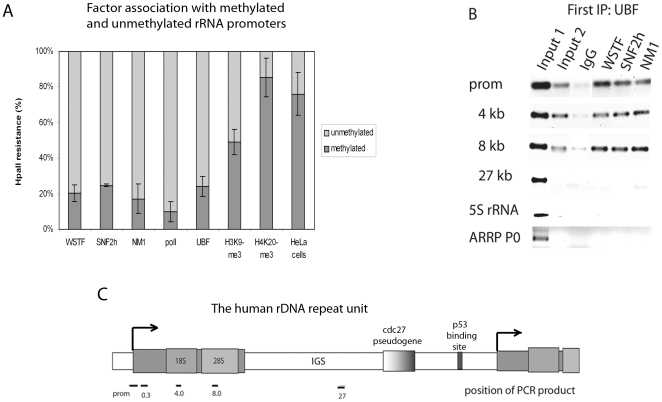
WSTF, SNF2h and NM1 associate with active rRNA genes. (**A**) HpaII and MspI digestion of DNA from ChIPs precipitated with antibodies against WSTF, SNF2h and NM1, RNA pol I, UBF, H3K9-me3 and H4K20-me3 as indicated. “HeLa cells” represents HpaII digestion of the input sample. The degree of methylation was correlated to the level of PCR product produced with a primer pair over the promoter. 100% represents 100% methylated promoter-DNA (not digested DNA), 0% represents no methylation of the promoter (the signal observed in digestions with MspI). Error bars represent standard deviations of five separate experiments. (**B**) Sequential ChIP using an UBF antibody in the first IP, and WSTF, SNF2h and NM1 and IgG antibodies as indicated in the second IP. Input 1 represents 5% of the chromatin used in the IP, input 2 represents 10% of the material released from the beads. The primer pairs used are shown in Fig. 1C, in addition to the 5S rRNA, and at the ARRP P0 gene as indicated. (**C**) Schematic image of the human rDNA repeat unit, with the position of the PCR product formed from the primer pairs for the ribosomal gene repeat.

The WSTF and NM1 localise to the fibrillar region in the nucleoli, where transcription takes place, suggesting a role at active gene copies [20], [31]. The three B-WICH core components, the WSTF, SNF2h and NM1 proteins, are also found at promoters and in the transcribed regions of rRNA genes, and they are required for increased RNA pol I transcription [20]. In the present study, we examined biochemically whether the proteins were associated with the active or inactive gene copies by chromatin immunoprecipitation (ChIP) followed by digestion of the DNA by methylation-sensitive HpaII. The B-WICH components exhibited a binding pattern more similar to that of the RNA pol I, and the activator UBF than to that of the silencing histone mark H4K20-me3 (Figure 1A). The methylation state of the ribosomal gene promoter in human cells is more complex than that in mouse cells, and human rDNA still retains methylations on CpGs in the active promoter [32]–[34]. The silencing histone mark H3K9-me3 also bound to methylated and unmethylated rRNA promoters to an equal degree, indicating that H3K9-me is found in active chromatin [24]. We therefore performed sequential ChIP using an UBF antibody to examine the degree of co-localisation between UBF and B-WICH factors on the same DNA fragment. WSTF, SNF2H and NM1 were co-immunoprecipitated with UBF at promoter DNA and at DNA sites in the transcribed region, in the 18S (4 kb) and 28S (8 kb) part of the gene (Figure 1B), demonstrating that all three proteins associate with active UBF-bound genes. No UBF was detected in the intergenic spacer (IGS) at a position 27 kb from transcription start (see Figure 1C for the schematic structure of the human rRNA gene repeat), to the 5S rRNA genes or the gene for the ribosomal protein ARRP0, transcribed by RNA pol III and RNA pol II, respectively.

### WSTF knock down results in chromatin changes and reduced RNA pol I factor binding

**Figure 2:**
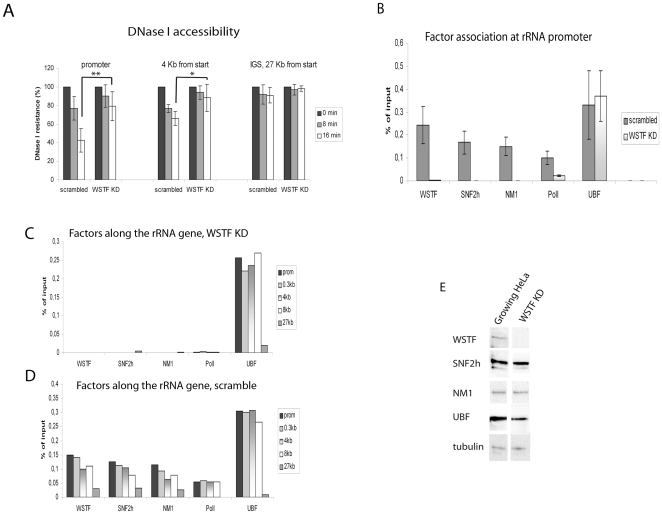
WSTF alters the chromatin structure at the rDNA and changes factor access to DNA. (**A**) DNAse digestion of cells for the times indicated, before preparing DNA and amplifying regions at the rRNA promoter, 4 kb into the gene, and in the IGS (at position 27 kb from transcription start site) with specific PCR primer pairs. The signal intensities of the products were quantified using Quantity One, Bio-RAD. Error bars represent standard deviation **: p-value of 0.0007, *: p-value of 0.04 (Student t-test, paired, n = 4). (**B**) ChIP of WSTF KD cells and cells transfected with scrambled control SiRNA using primers over the rRNA promoter to detect DNA precipitated by the antibodies indicated below the bars. The values are presented as the percentage of the input signal for each primer pair. Error bars represent standard deviations from four separate experiments. (**C**) ChIP of WSTF KD cells using PCR primers which detected the position along the rDNA repeat as indicated with the antibodies as indicated below the bars. The signals are the percentages of the input signal for each primer pair. One representative experiment of four. (**D**) ChIP of cells transfected with scrambled control SiRNA using PCR primers which detected the position along the rDNA repeat as indicated with the antibodies as indicated below the bars. The signals are the percentages of the input signal for each primer pair. One representative experiment of four. (**E**) Immunoblots (7% SDS-PAGE) of proteins from cell lysates from growing HeLa cells and WSTF KD cells probed with the antibodies indicated at the left.

To examine the role of B-WICH in the transcription of ribosomal genes, we assessed the chromatin structure over the 47/45S rRNA genes in cells in which WSTF had been knocked down for 36 hours. WSTF knock down (WSTF KD) cells, and cells transfected with scrambled SiRNA, were digested with DNAse I, samples were taken at different times and the levels of undigested DNA over the promoter, in the transcribed region (the 18S region) and the IGS (position 27) were detected by PCR. The DNA at the promoter in WSTF knock down cells was more protected than that in cells transfected with scrambled SiRNA (Figure 2A) (p = 0.0007 after 16 min, Student t-test). The DNase I sensitivity in the transcribed region, detected by the primer pair 4 kb, was less affected by WSTF knock down (p = 0.04), and no effect was observed in the IGS (Figure 2A).

The effect of WSTF knock down on the chromatin structure at the rRNA gene promoter prompted us to examine the recruitment of factors to rDNA in WSTF KD cells. Binding of the factor UBF to the rRNA promoter alters the chromatin structure, which allows SL1 and the RNA pol I to bind and initiate transcription [35]. However, no change in the level of UBF associated with the promoter (Figure 2B) or along the gene (Figures 2C and 2D) was detected in WSTF KD cells compared to the level associated in cells transfected with scrambled SiRNA. In contrast, WSTF knock down reduced the binding of SNF2h and NM1 at the promoter (Figure 2B) and along the gene compared to the binding pattern in control cells transfected with scrambled SiRNA (Figures 2C and 2D). The absence of SNF2h and NM1 at the rDNA in WSTF KD cells was not caused by a decrease in the protein levels (Figure 2E). The level of UBF was reduced in WSTF KD cells (Figure 2E), but this was not reflected in the binding to the gene. These results suggest that B-WICH is required for an open chromatin structure at the rRNA promoter, but not by preventing UBF binding to the rRNA.

We also investigated the effect of WSTF KD on rRNA-processing intermediates, since the B-WICH complex contains RNA-processing proteins, such as RNA helicaseII/Guα/DXX21 [21]. In contrast to the effect on chromatin structure, knock down of the WSTF protein did not have a detectable effect on the rRNA intermediates compared with cells transfected with control shRNA (Figure S1). However, a decrease in overall formation of 45S pre-rRNA was observed, indicating that the effect of the B-WICH is to activate or maintain ribosomal transcription.

### WSTF knock down results in changed histone modifications at the rDNA loci

**Figure 3:**
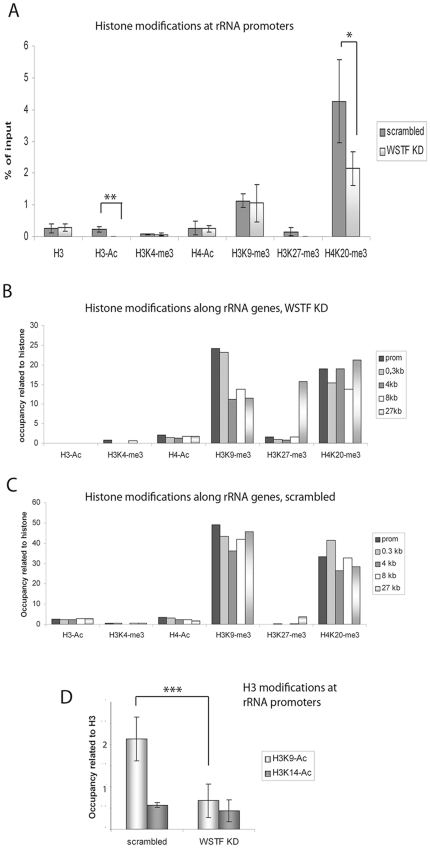
H3-Ac levels are reduced in WSTF KD cells. (**A**) ChIP of WSTF KD cells and scrambled control cells at the rRNA promoter with the antibodies indicated below the bars. The values are presented as percentages of the input signal. Error bars represent standard deviations from four separate experiments. **: p-value = 5.5×10^−5^, *: p-value = 0.06 (Student t-test, two-sample equal variance). (**B** and **C**) ChIP of WSTF KD cells (B) and scrambled control cells (C) with the antibodies indicated below the bars, detecting the positions along the rDNA repeat by PCR primers as indicated. The values presented have been adjusted to the signal for the relevant histone for each primer pair and they are presented for one representative experiment of four. (**D**) ChIP of WSTF KD cells and scrambled control cells using specific H3K9-Ac and H3K14-Ac antibodies, as indicated below the bars. Error bars represent standard deviations from four separate experiments. ***: p-value = 0.0017(Student t-test, two-sample equal variance).

Histone modifications have been observed on active rRNA genes and can contribute to the regulation of rRNA transcription. We first investigated the effect of WSTF knock down on the states of histone modifications. The level of histone modifications was determined by quantifying nuclear stainings of cells in which WSTF had been knocked down (Figure S2A) and these showed that the levels of histone acetylations were lower in WSTF KD cells than in scrambled control cells (Figure S2B). The reduction in acetylated histones was confirmed by immunoblots of chromatin fractions from cells transfected with SiWSTF or scrambled SiRNA (Figure S2C). The effect of WSTF knock down on the global acetylation levels led us to investigate whether the B-WICH complex changes the histone acetylation pattern at the rDNA loci. WSTF KD resulted in a reduced level of H3-Ac at rRNA promoters compared with the level in cells transfected with scrambled SiRNA (p = 0.000055, Student t-test) (Figure 3A). Other active histone marks tested, H4-Ac and H3K4-me3, were not affected, and present at similar levels as in scrambled control cells (Figure 3A). The silencing mark H4K20-me3 was reduced in WSTF KD cells (p = 0.06), whereas the mark H3K9-me3 was not. H3-Ac was not found along the gene in WSTF KD cells, but the distributions of other active histone marks were similar to those found in scrambled control cells (Figures 3B and 3C).

We next investigated whether the down regulation in acetylation was attributed to one specific lysine in the H3 tail by using specific antibodies. The level of H3K9-Ac was more affected at the promoter than that of H3K14-Ac in WSTF KD cells (Figure 3D), and was reduced 3.1-fold (p = 0.0017) compared to the H3K9-Ac level in cells transfected with scrambled SiRNA. The level of H3K14-Ac was not significantly different in WSTF KD cells from that in control cells transfected with scrambled SiRNA.

### The binding of B-WICH to rDNA is reduced in serum-starved cells

**Figure 4:**
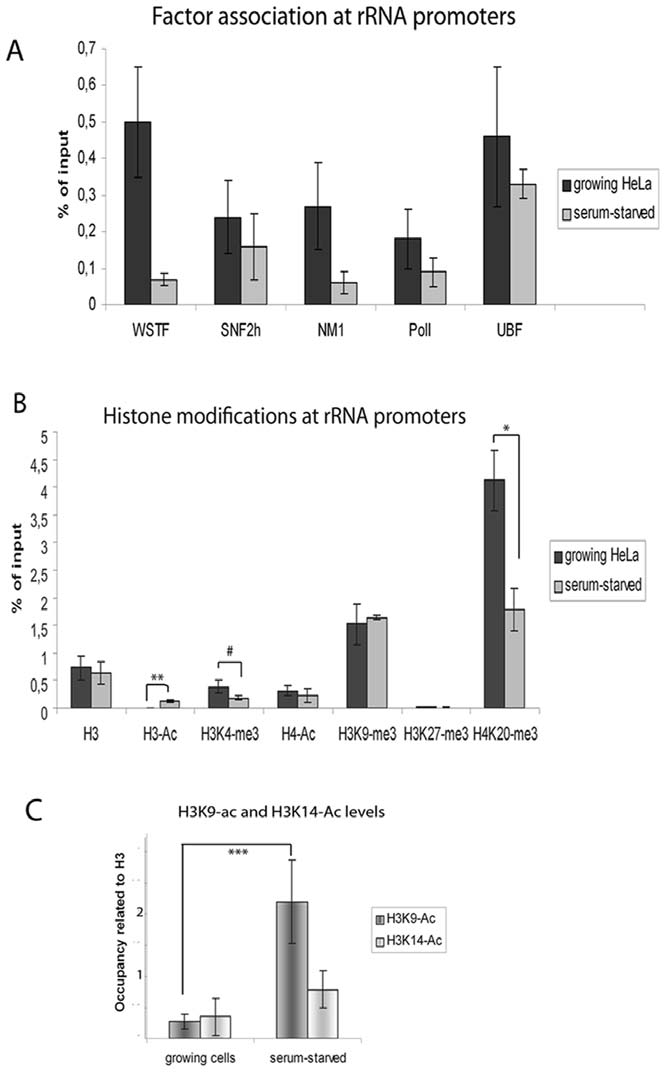
H3-Ac levels are reduced in growing HeLa cells. (**A**) ChIP of growing cells and serum-starved cells with antibodies as indicated below, detected at the rRNA promoter. The values are presented as the percentages of the input signal for each primer pair. Error bars represent standard deviations from four separate experiments. (**B**) ChIP of growing HeLa cells and serum-starved cells at the rRNA promoters with antibodies indicated below the bars. The values are presented as the percentage of the input signal for each primer pair. Error bars represent standard deviations from six separate experiments. **: p-value = 0.0001, *: p-value = 0.01, #: p = 0.05 (Student t-test, two-sample equal variance). (**C**) ChIP of growing cells and of serum-starved cells using the antibodies indicated detecting the rRNA promoter. The signals have been adjusted to the signal from histone H3 in each experiment. Error bars represent standard deviations from four separate experiments. ***: p-value = 0.00036 (Student t-test, two-sample equal variance).

The effect of WSTF knock down on H3K9-Ac prompted us to investigate whether the H3K9-Ac was involved in regulating rRNA transcription in HeLa cells. We took advantage of serum starvation, which gives non-proliferating cells with a reduced rRNA synthesis [36]. Serum starvation for 72 hours had an impact on the binding of B-WICH factors to the promoter, as the bindings of WSTF and NM1 were clearly reduced (Figure 4A). The protein level of WSTF was also reduced in serum starved cells, whereas the levels of NM1 and SNF2h remained unaltered in serum-starved cells (Figure S3A). RNA pol I also displayed a slight reduction in binding in serum-starved cells (Figure 4A), reflecting the lower transcription level (Figure S3B). However, serum starvation did not have any effect on the level of the association of UBF at the promoter (Figure 4A).

### Histone acetylation at the promoter is low in growing cells

Several studies have shown that histone acetylations, which are associated with active RNA pol II genes, are correlated with the regulation of ribosomal transcription. It has been shown that H4-Ac is deacetylated by NoRC upon silencing, and the level of H3-Ac changes in response to glucose [16]. Despite a higher level of transcription in growing HeLa cells (Figure S3B) (50% confluent), the H3-Ac level associated with the rRNA promoter was lower than it was in serum-starved cells (p = 0.0001), without any change in the level of histone H3 (Figure 4B). This did not follow the pattern in RNA polymerase II transcription, where H3-Ac signals an open chromatin structure over the promoter and a high transcription level [37]. The active histone mark, H4-Ac was unaltered, whereas H3K4-me3 was slightly reduced in serum-starved cells (p = 0.05) (Figure 4B). The lower level of H3K4-me3 is consistent with a reduced transcription rate. The level of H4K20-me3 at the promoter was also reduced in serum-starved cells compared to growing cells (p = 0.01) (Figure 4B). The distribution of histone marks along the gene is shown in Figure S4.

We also investigated the specificity of the acetylated site on histone H3, H3K9 or H3K14, in growing cells and serum-starved cells. A clearly reduced level of H3K9-Ac was detected in growing cells, compared with the level found in serum-starved cells (p = 0.00036) (Figure 4C). The H3K14-Ac level, on the other hand, was not significantly different in the two samples. These results indicate that H3K9-Ac at the ribosomal genes is more sensitive to external signalling events causing transcriptional changes.

### WSTF KD results in a lower recruitment of HATs at the rDNA

**Figure 5:**
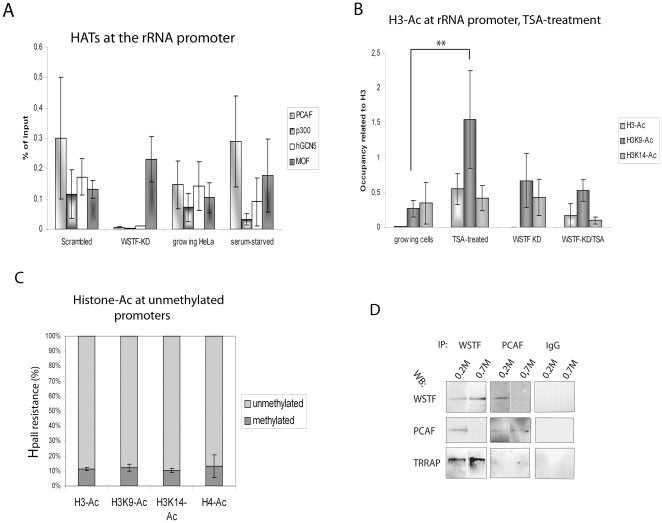
WSTF allows for the association of several HATs with rRNA promoters. (**A**) ChIP of scrambled control cells, WSTF KD cells, growing cells and serum-starved cells with the antibodies indicated and PCR signals were measured at the rRNA gene promoter. The values are presented as percentages of the input signal. Error bars represent standard deviations from four separate experiments. (**B**) ChIP of growing cells, TSA-treated cells (4 hours), WSTF KD cells and WSTF KD cells treated with TSA (4 hours) using the antibodies indicated. The signals have been adjusted to the signal from histone H3 in each experiment. Error bars represent standard deviations from four separate experiments. **: p-value = 0.0036 (Student t-test, two-sample equal variance). (**C**) HpaII and MspI digestion of DNA from ChIPs precipitated with antibodies against H3-Ac, H3K9-Ac, H3K14-Ac and H4-Ac. The degree of methylation was correlated to the level of PCR product produced with a primer pair over the promoter. 100% represents 100% methylated promoter DNA, 0% represents the level observed in digestions with MspI. (**D**) Immunoblot (10% SDS-PAGE) of immunoprecipitations using antibodies against WSTF and PCAF, as indicated, of nuclear extracts prepared at 0.2 M KCl and 0.7 M KCl. Co-immunoprecipitated proteins were detected with the antibodies marked at the left. IgG was used as a control.

Several histone acetyl-transferases (HATs) have been found at the rDNA promoter [19] and the low levels of H3-Ac in growing cells and in WSTF KD cells led us to investigate the occupancies at the rRNA promoter of HATs implicated in H3 acetylation: PCAF, GCN5, and p300. The occupancies of PCAF, GCN5 and p300 were reduced in WSTF KD cells, whereas these proteins were found at the ribosomal promoter in cells transfected with scrambled SiRNA, in growing cells, and serum-starved cells (Figure 5A). In contrast to the histone H3-specific HATs, the MOF protein, which is implicated in H4 acetylation [38] and acetylation of the TIP5 protein in ribosomal gene silencing [39], was present at the promoter in WSTF KD cells, as it was in scrambled cells, growing cells and serum-starved cells (Figure 5A). We conclude that the lack of H3-Ac in WSTF KD cells is a result of a reduced binding of histone H3-specific HATs at the promoter. The low levels of H3-Ac in growing cells could not be explained by a reduced level of HATs at the promoter, suggesting that the underlying mechanism is different from the mechanism that operates in WSTF KD cells. To further investigate the mechanism in growing cells, we first established whether acetylated H3 was formed on rDNA in growing HeLa cells. We treated cells with trichostatin A (TSA) for 4 hours before preparing chromatin for ChIP. TSA inhibits histone deacetylases and produces a hyperacetylated state of chromatin [40]. The level of H3-Ac, in particular H3K9-Ac (p = 0.0036), was elevated in growing cells treated with TSA (Figure 5B). WSTF KD cells treated with TSA did not show an increase in the level of H3K9-Ac, and only a small increase of H3-Ac. By using the HpaII resistance assay, we showed that the histones harbouring the acetylation in TSA-treated growing HeLa cells were associated with the active gene copies (Figure 5C). We conclude that the low levels of acetylated histones found in growing and WSTF KD cells are a result of different mechanisms; reduced association of H3-specific HATs in WSTF KD cells and a rapid turn-over of H3-acetylation in highly proliferating cells by HDACs.

The finding that WSTF knock down abolished the binding of other B-WICH proteins and of several HATs to rDNA led us to examine direct interactions between WSTF and the HATs: PCAF, p300, and GCN5. We also investigated TRRAP, which interacts with two different HATs, GCN5 and Tip60. We used different salt concentrations: 0.2 M KCl and 0.7 M KCl, to extract nuclear proteins before the IP to detect interactions that were missed in the initial purification, performed at 0.7 M KCl [21]. Interactions between WSTF and the proteins PCAF and TRRAP were detected at low salt concentrations (Figure 5D). TRRAP also interacted with WSTF at 0.7 M KCl. hGCN5 and p300 did not interact with WSTF at any KCl concentration (Figure S5). These results suggest that the B-WICH not only remodels the chromatin but also facilitates the recruitment of specific HATs to the rDNA promoter. We conclude, however, that the direct recruitment is secondary because of the weak interaction.

### WSTF is required for opening the chromatin structure 200 bp upstream at the start site

**Figure 6:**
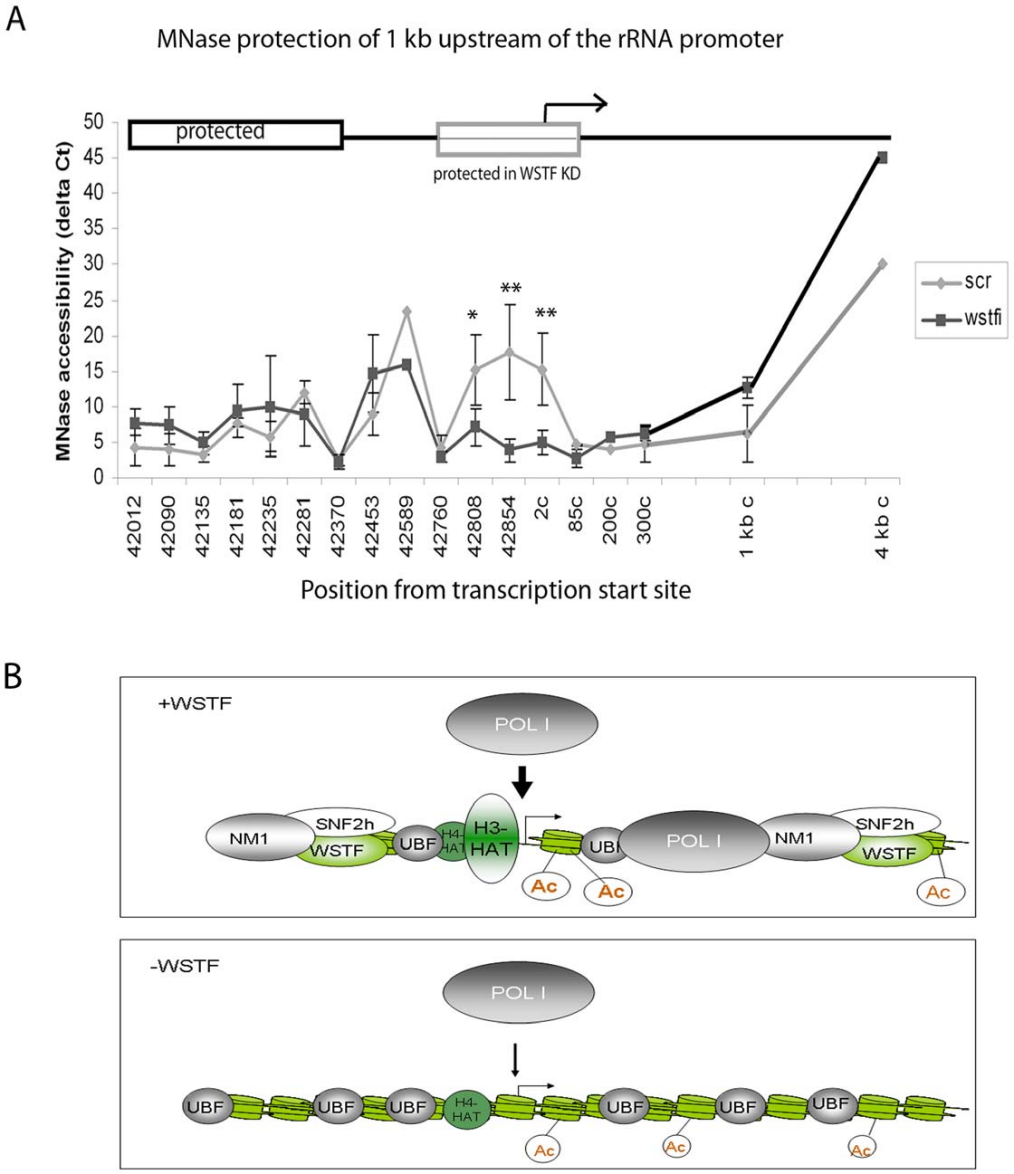
WSTF affects a 200 bp region at the rRNA promoter. **A**) The chromatin profile from WSTF KD cells (black line) and scrambled cells (grey line) presented as 2^ΔCt^ of undigested and MNase digested cross-linked chromatin The position of the primer pair used is given below the graph; 2c (coding) denotes position 2 in the coding region. Error bars represent standard deviations of three separate experiments. *: p = 0.05, **: p = 0.02 (Student t-test, two-sample equal variance). The graph is not drawn to proportion. The gene region is depicted above the graph, showing protected areas in boxes. The region specific to the effect of WSTF KD is marked with a grey box. The arrow indicates transcription start site. (**B**) Schematic model of the role of B-WICH in rRNA transcription. The B-WICH complex is required for the access of B-WICH factors and H3-specific histone acetyl-transferases to the DNA, resulting in H3K9-acetylation.

Our results suggest that the promotion of H3-Ac at the promoter by B-WICH is secondary to a chromatin remodelling event. We therefore investigated the changes in chromatin structure upstream of the transcription start site (−1 kb to +300 kb) upon WSTF knock down further by using a high-resolution MNase assay of cross-linked chromatin [41]. WSTF knock down caused a protection of approximately 200 bp over the promoter (Figure 6A), a region including the upstream control element (UCE) and the core promoter element (CORE). This region was resistant to MNase digestion, indicating that this region binds more proteins in WSTF KD cells. Cells transfected with scrambled SiRNA demonstrated a more open chromatin structure over this 200 bp region. WSTF knock down did not result in any major changes in the chromatin structure outside of this region. A MNase-sensitive site of 135 bp appeared both in scrambled and in WSTF KD cells at 400 bp upstream of the transcription start site (see schematic outline of the region in Figure 6A). The DNA is relatively protected upstream of the sensitive site in both scrambled and WSTF KD cells, a region containing conserved CpGs at a CTCF-binding site (position 42.1 kb from transcription start site) [42].

## Discussion

We show here that the B-WICH core, the WSTF-SNF2h-NM1 proteins, is required for altering the chromatin structure at the rDNA promoter and thereby allowing transcription to occur. We demonstrate that the remodelling caused by the B-WICH complex is restricted to a specific region 200 bp around the promoter, including the UCE (to which UBF associates), the core element (to which the pre-initiation complex associates) and the transcription start site. WSTF, as a component of the WICH complex, is involved in the spacing of nucleosomes during replication and, as a result of chromatin remodelling, affects transcription globally by decreasing H3K9-me2 and thereby the recruitment of heterochromatin proteins [43]. We show here that WSTF also affects the level of histone acetylation globally. In line with these findings, the presence of WSTF leads to increased H3-Ac at the rRNA genes. Two ATP-dependent chromatin-remodelling complexes, CSB and B-WICH, are associated with the ribosomal gene repeat. The CSB protein remodels chromatin by recruiting histone methyl-transferase G9a, inducing H3K9-me [24]. Instead of changing the methylation pattern of histone H3 at the rDNA genes, the B-WICH alters the H3 acetylation pattern, allowing for increased levels of H3K9-Ac. Interestingly, CSB is an interacting partner with the B-WICH complex [20], while none of the other proteins identified in the CSB IP/150 complex associate with the B-WICH core. This could mean that the function of the two complexes is coordinated, possibly responding to different activating pathways. The finding that the action of B-WICH regulates histone acetylation suggests that RNA pol I and RNA pol II transcription use similar mechanisms to regulate chromatin structure and gene expression, in addition to gene-specific mechanisms.

The B-WICH subunits associated mainly with unmethylated rDNA, and were present together with UBF at the promoter and in the coding region. The methylation pattern of the promoter is complex in human cells [32]–[34], with UBF associated to methylated CpGs [32], [33]. It is unclear whether these genes are active or inactive [32], [33]. In addition, the histone mark H3K9-me, which is believed to be present on silent copies, is also present on active gene copies [24]. Histone H3K9-me3 in our HpaII-cleavage assay displayed a pattern that suggested that H3K9-me3 is not confined to silent gene copies. We suggest that the B-WICH follows UBF, and is present mainly at active gene copies.

Histone modifications regulate rRNA transcription, in particular in the silencing of rRNA genes. Histone acetylations, both of H3 and H4, have been connected with active genes [11], [12], [16], [39], and we now link H3 acetylation to chromatin-remodelling events. The association of several HATs was abolished at the rRNA gene in WSTF KD cells, correlated to a reduced level of H3K9-Ac. Interestingly, H4-Ac, a modification that has been coupled to the regulation of RNA pol I transcription, was not affected. This could be explained by the finding that MOF was present at the promoter also in WSTF KD cells, indicating that WSTF-SNF2h chromatin remodelling has specificity for HATs with H3K9-Ac-activity. MOF associates with TIP5 and regulates the gene-silencing activity of NoRC by direct acetylation of TIP5 [39]. Since WSTF knock down did not alter the association of MOF with the RNA gene promoter, it is not likely that the B-WICH complex interferes with NoRC activity. We propose instead that B-WICH is required for the regulation of RNA pol I transcription in different cellular response pathways, such as responses to serum starvation and glucose starvation. The H3-Ac levels, or H3K9-Ac levels, at the rRNA promoter are decreased in metabolically deprived cells, concomitant with a reduced 45S pre-rRNA transcription [16], [39]. The specific effect conferred by the B-WICH complex on the H3K9-Ac level could be achieved by direct recruitment, as WSTF interacted with PCAF and TRRAP directly. However, the interactions to the HATs tested were weak, or for p300 and GCN5 not detected, suggesting that direct interactions is not the main mechanism responsible for the increased H3 acetylation. The interactions may facilitate the recruitment of complexes containing PCAF and of the TRRAP-complex (which includes GCN5) to the gene. We have not yet identified the HAT that is specific for the acetylation of H3K9, and all three HATs investigated here: PCAF, p300 and GCN5, have H3K9 acetyl-transferase activity [44]. We propose that the mechanism by which the B-WICH operates at the rDNA locus is to actively prevent compaction of chromatin and thereby allow and facilitate for HATs to be recruited, resulting in acetylated histone H3, specifically H3K9-Ac (see Figure 6B). The lack of NM1 at the rDNA in WSTF KD cells could further contribute to the inhibition of RNA pol I transcription. NM1 has been suggested to act as a molecular switch contacting nuclear actin attached to RNA pol I in active transcription [31], [45], [46].

In addition to the effects of B-WICH, we found no or a very low level of H3-Ac present at the rRNA promoter in growing HeLa cells, despite the fact that several HATs were present. This could be the result of nucleosomes harbouring H3-Ac being easily disrupted due to a very high transcription rate, or HDACs being recruited to the active copies to prevent promiscuous initiation of transcription. Treating growing cells with TSA increased the level of H3-Ac at the actively transcribed promoters, suggesting that the low level of H3-Ac at the promoter is caused by the presence of HDACs, recruited to prevent promiscuous transcriptional initiation. We cannot totally exclude that the low levels of H3-Ac are partially explained by the disruption of nucleosomes harbouring H3K9-Ac, however, since H3-Ac appears in serum-starved cells with a slower RNA pol I transcription rate. It has been suggested that positioned nucleosomes in the rRNA gene promoter set the silent state, and chromatin remodelling is required to activate transcription by changing the position of the nucleosomes [47]. Our results support the finding that nucleosome changes at the promoter contribute to active transcription. WSTF did not affect the association of the chromatin-remodelling protein UBF to the rRNA, although the overall protein level decreased. However, the protein level may change within certain limits without affecting transcription [48]. The alteration of the chromatin structure upon WSTF depletion, and the lack of any change in UBF association, suggest that nucleosomes are involved in the regulation of rRNA synthesis. In *S. cerevisiae*, no histones [49] or non-canonical histones are present along the transcribed genes [50]. Active gene copies in mammalian cells are also sensitive to psoralen cross-linking, demonstrating that these genes have a more open chromatin structure [3]. This could be taken as evidence that mammalian cells have a structure similar to that found in *S. cerevisiae*. Recently, however, the histone chaperone complex FACT was found to function in RNA pol I transcription in human cells, indicating that there is a role for histones in RNA pol I transcriptional elongation in mammalian cells [51]. Our results show that histones are present at the rDNA, and contribute to the chromatin structure.

The WSTF gene is one of the genes deleted haploinsufficiently on chromosome 7 in William syndrome, a developmental and mental disorder [52]. Our finding that WSTF in the B-WICH complex affects the chromatin structure in the nucleolus and ribosomal transcription suggests a role of the WSTF in cell growth and proliferation. We have also shown that the B-WICH complex is involved in RNA pol III transcription [21], which further implies the role of B-WICH in growth processes. It is difficult, however, to assess the role of the separate WSTF-containing complexes in the development of the syndrome, since they are involved in several processes: replication, DNA repair and vitamin D-mediated transcription.

WSTF was required for the integrity of the B-WICH core complex at the rRNA gene promoter as knock down of WSTF resulted in reduced promoter occupancy of the B-WICH subunits SNF2h and NM1. It is less clear what recruits the WSTF to the ribosomal genes. WSTF does not contain a DNA-binding domain and is recruited to replication foci by binding to PCNA [43]; it can also bind to H3K14-Ac [53]. TIP5 in NoRC associates with the rDNA by interacting with H4K16-Ac, TTF-1 and pRNA [54]. It is possible that the level of H3K14-Ac at the rRNA promoter recruits WSTF via its bromodomain. The WSTF, as well as TIP5, belongs to the WAL/BAZ family of proteins, whose members share a characteristic domain structure [55]. Apart from a bromodomain that binds acetylated proteins, these proteins also harbour a PHD Zn-finger domain. Other proteins in this family, such as BPTF in the ISWI-containing NURF complex, bind methylated histones [56]. PHD domains in other proteins also display affinity for methylated histones, such as PHF8, which binds to H3K4-me3 in rDNA [57]. It is tempting to speculate that WSTF also binds to rDNA via its PHD domain and this interaction is further potentiated by its binding to H3K14-Ac through its bromodomain. This would then lead to an alteration of the chromatin structure and a higher level of H3K9-Ac by the recruitment of specific HATs in response to external stimuli.

## Methods

### Antibodies used

WSTF, SNF2h, MOF, H3, H3K9-Ac, H3K14-Ac, H3K4-me3, H3K9-me3, H4, H4K20-me3 were from Abcam, H3-Ac and H4-Ac were from Millipore, PCAF was from Abnova and Santa-Cruz, GCN5 from Abcam and from Abnova, and p300 was from Abnova and Abcam, TRRAP was from Bethyl Laboratories and Santa Cruz, RNA pol I was a kind gift from T. Moss, and UBF was from Santa Cruz. Trichostatin A (TSA) and L-α-lysophosphatidylcholine were purchased from Sigma-Aldrich and used at a concentration of 300 ng/ml for 4 hours and 0.01%, respectively.

### Cell lines

Adherent HeLa cells (originally purchased from ATCC, US) were cultivated in 10% FCS in DMEM medium supplemented with PEST at 10% CO_2_.

### Chromatin immunoprecipitations (ChIP)

ChIP analyses were performed as described in Cavellán et al. [21] based on Takahashi et al. [58]. The IP was performed in 1% Triton-100 and 0.1% DOC, to which 15 µl protein A/G was added. The IP was subsequently washed five times with RIPA buffer containing NaCl. The primers used for detecting DNA sequences are given in Table S1.

Re-ChIP was performed as described by Young et al. [59]. Briefly, the first antibody-antigen interaction was disrupted by adding 10 mM DTT, final concentration. The DTT was diluted 50-fold prior to addition of the second antibody.

qPCR was performed using SYBR-green from KAPA according to the manufacturer's instructions. The primer concentration was 2.5 µM and the samples analysed by Rotor-Gene 6000 series software 1.7. The PCR conditions were: hold 95°C for 3 minutes, followed by cycles of 95°C for 3 seconds, 60°C for 20 seconds, 72°C for 3 seconds. The results were analysed using an average of Ct of No antibody and IgG as background. The 2^ΔCt^ of each sample in triplicates was then related to the 2^ΔCt^ of the input sample. When indicated, the 2^ΔCt^ of histone modification was related to the relevant 2^ΔCt^ of core histone.

### HpaII digestion

ChIP samples were digested with HpaII and MspI in separate tubes, and the samples analysed by qPCR, using promoter primers and the 18 kb primer pair as a control.

### DNaseI digestions

Cells were treated with LPC buffer (0.01% L-α-lysophosphatidylcholine, 150 mM sucrose, 80 mM KCl, 35 mM Hepes at pH 7.4, 5 mM K_2_HPO_4_, 5 mM MgCl_2_, 0.5 mM CaCl_2_) for 90 seconds. Cells were treated with 1 U DNaseI in DNase-buffer (20 mM Tris-HCl at pH 7.5, 60 mM KCl, 15 mM NaCl, 250 mM sucrose, 1 mM CaCl_2_, 1 mM DTT and protease inhibitors) for the times indicated, then stopped by adding 3.3 mM EDTA. The samples were treated with proteinase K, 1% SDS was added, and the DNA was extracted with phenol:chloroform. The samples were digested with 10 U of EcoRI and 10 U of HindIII, and were analysed by PCR over the region indicated. The intensity of the product was quantified using Quantity One, Bio-Rad.

### Immunoprecipitations

Cells were homogenized in hypotonic homogenizing buffer (20 mM HEPES at pH 7.9, 10 mM KCl, 1.5 mM MgCl_2_, 1 mM DTT supplemented with 0.5 mg/ml each of aprotinin, antipain, chymostatin and leupeptin, 10 µM benzamidine, 1 µg/ml phenantroline, 5 µg/ml pepstatin A and 1 mM PMSF), using 30 strokes in a Dounce homogenizer, pestle B. Nuclear extract was then prepared as described by Ryme et al. [60] using 200 and 700 mM KCl in the lysis buffer. The HeLa nuclear extract (200 to 300 µg) was incubated with antibody over night. Proteins-antibody complexes were precipitated using Protein A/G beads. The samples were boiled in sample buffer and separated on a 10% SDS-PAGE. Cell lysates were prepared using 0.42 M NaCl, as described in Ryme et al. [60], when not indicated otherwise.

### High-resolution MNase assay

Cells were cross-linked with 1% formaldehyde for 20 minutes and chromatin prepared as for ChIP, but washed with buffer D (25% glycerol, 5 mM Mg-acetate, 50 mM Tris at pH 8.0, 0.1 mM EDTA, 5 mM DTT) as described in Petesch and Lis [41]. The chromatin was sonicated lightly in MNase buffer (60 mM KCl, 15 mM NaCl, 15 mM Tris at pH 7.4, 0.5 mM DTT, 0.25 M sucrose, 1.0 mM CaCl_2_), 8 times for 30 seconds, before digestion. The equivalent of 0.4×10^6^ cells was used in each reaction, and the level of DNA was first adjusted to be in the same range in the samples from all different treatments. Two samples from each treatment were used: 0 U MNase and 20 U MNase, and the reactions were performed at 37°C for 30 minutes. The reactions were stopped by adding 12.5 mM EDTA/0.5% SDS. The samples were treated with protein K for 3 h, followed by reversal of the cross-linking at 65°C for at least 5 hours. DNA was extracted as described for ChIP by Cavellán et al. [21]. The digest was evaluated by qPCR using the primer pairs, giving a product of approximately 100 bp, given in Table S2. The results were analysed by calculating ΔCt between the reaction with no MNase and the digested reaction. The values are presented as 2^ΔCt^. During the optimalisation, several different MNase concentrations were used, and 2 U MNase did not give a difference in digestion from uncut control chromatin. Chromatin from scrambled and untransfected cells gave the same MNase digestion pattern.

## Supporting Information

Figure S1 **B-WICH does not affect rRNA processing but pre-rRNA transcription.** (**A**) Total RNA from WSTF KD HeLa cells and control cells transfected with scrambled pSuper vector was prepared and 15 µg of each sample was run on a 1% agarose-gel. The RNA was transferred to a nitrocellulose membrane and probed with radioactively labelled oligonucleotides specific for processing products: ETS-1 for processing to 18S: ITS-2 (and ITS-1) for processing to 28S [61]. (**B**) The signals were quantified in a Fuji Phosphoimager, and the ratio between products calculated for each sample. (**C**) The ratio between the 45S pre-rRNA signals from WSTF KD cells and scrambled control cells were also calculated. No difference was detected between the processing intermediates and the 45 pre-rRNA levels in control cells and WSTF KD cells, indicating that no accumulation of intermediates occurs (see B). However, when the levels of 45S pre-RNA were compared, lower levels were found in the WSTF KD cells (see Figure S1C).
(TIF)

Figure S2 **WSTF increases histone acetylation globally.** (**A**) Immunolocalisations of WSTF and modified histones. Cells were fixed for 15 min with formaldehyde 3.7% at room temperature, permeabilised with Triton X-100 0.5% for 7 min, and blocked with 0.5% milk for 30 min. Primary antibodies H3-Ac, H4-Ac, H3K9-Ac, H3K9-me3, H4K20-me3, and WSTF were incubated for one hour, and the secondary antibodies coupled either to Cy2 or Alexa 568 (Jackson, Invitrogen) for 45 min at RT. DNA was revealed by DAPI staining (300 nM for 3 min at RT) and coverslips were mounted with Mowiol (Merck). Images were obtained with a confocal microscope (Zeiss LSM 510 meta) with 63X oil objective of NA 1.3. Localisation of histones (green) and WSTF (red) in control cells (left panels, scrambled cells) or after silencing of WSTF expression (right panels, WSTF KD cells). The chromatin is revealed by DAPI labelling (blue). Scale bar, 10 µm. The upper panel shows the localisations of acetylated forms of histones H3-Ac, H4-Ac and H3K9-Ac (upper, middle and lower row). The lower panel shows the localisations of methylated histones H3K9-me3 and H4K20-me3 (upper and lower row). (**B** and **C**) Quantification of fluorescence signals obtained after immunostaining histones, scrambled control cells and WSTF-silenced cells. Micrographs were analysed with ImageJ; the mean gray value of an individual nucleus was registered. The mean gray values were averaged and expressed as percentages. The average mean gray value of the controls was set as 100% of signal and the average of the mean gray values measured after silencing of WSTF expression was expressed proportionally. In each experiment the number of nuclei measured was between 45 and 74. Error bars represent standard deviations. (**D**) Immunoblots (15% SDS-PAGE) of nuclear extracts from WSTF KD cells and scrambled control cells. The antibodies used are indicated to the left. Histone H3 is used as a loading control.
(TIF)

Figure S3 **The 45S rRNA level and the WSTF protein level are reduced in serum-starved cells.** (**A**) Immunoblot (7% SDS-PAGE) of 30 µg of cell lysates (0.7 M KCl) from growing cells, serum-starved cells and re-fed cells (9 h), using antibodies against WSTF, SNF2h, and NM1, as indicated at the left. Actin was used as a loading control. (**B**) 45S rRNA levels in growing cells and in serum-starved cells, detected from reverse transcriptase (Invitrogen) converted RNA preparations, and amplified with primer pair 0.3 kb (see Fig. 1C). Primer pair detecting actin was used as a control.
(TIF)

Figure S4 **Growing cells have low levels of H3-Ac along the rRNA gene. (A**) ChIP of growing cells (upper panel) and ChIP of serum-starved cells (lower panel) with antibodies indicated below, in which PCR primers at the positions along the rDNA repeat as indicated were used. One representative experiment out of six is presented with the values adjusted to the signal for the relevant histone for each primer.
(TIF)

Figure S5 **GCN5 and p300 does not interact directly with WSTF.** Immunoblot (10% SDS-PAGE) of immunoprecipitations using antibodies against WSTF, GCN5 and p300, as indicated, of nuclear extracts prepared at 0.2 M KCl and 0.7 M KCl. Co-immunoprecipitated proteins were detected with the antibodies marked at the left. IgG was used as a control.
(TIF)

Table S1 **Primers used in the study (based on the human rRNA gene repeat, U13369).**
(DOC)

Table S2 **Primers used in the high resolution MNase assay, investigating 1 kb upstream of the transcription start site.**
(DOC)
