# Supplementary figures and images for: The Chromatin Remodelling Complex B-WICH Changes the Chromatin Structure and Recruits Histone Acetyl-Transferases to Active rRNA Genes
Source: PLoS One. 2011 Apr 29;6(4):e19184. doi: 10.1371/journal.pone.0019184 (PMC3084792; doi:10.1371/journal.pone.0019184)

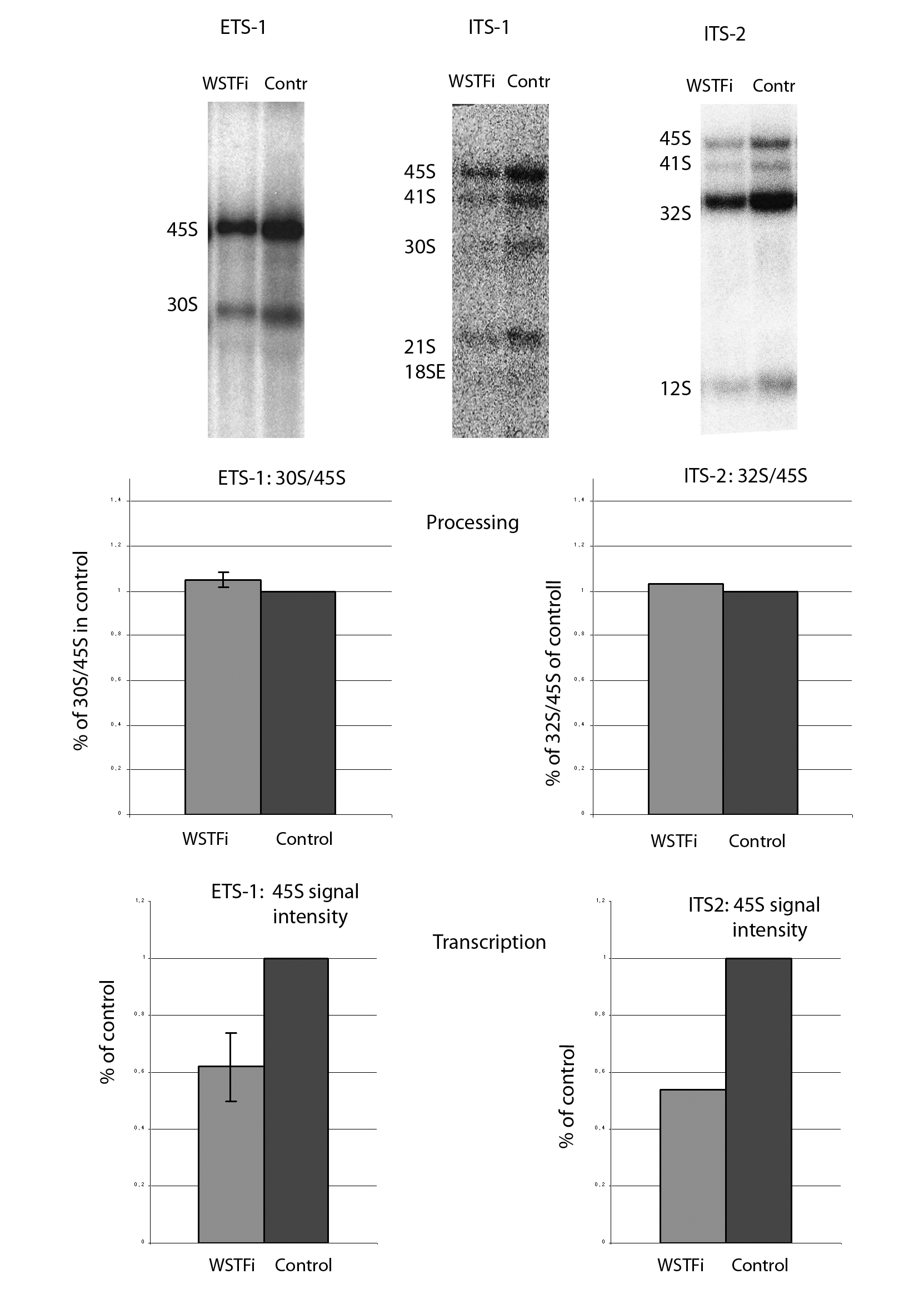

Supplement: Figure S1 — B-WICH does not affect rRNA processing but pre-rRNA transcription. (A) Total RNA from WSTF KD HeLa cells and control cells transfected with scrambled pSuper vector was prepared and 15 µg of each sample was run on a 1% agarose-gel. The RNA was transferred to a nitrocellulose membrane and probed with radioactively labelled oligonucleotides specific for processing products: ETS-1 for processing to 18S: ITS-2 (and ITS-1) for processing to 28S [61]. (B) The signals were quantified in a Fuji Phosphoimager, and the ratio between products calculated for each sample. (C) The ratio between the 45S pre-rRNA signals from WSTF KD cells and scrambled control cells were also calculated. No difference was detected between the processing intermediates and the 45 pre-rRNA levels in control cells and WSTF KD cells, indicating that no accumulation of intermediates occurs (see B). However, when the levels of 45S pre-RNA were compared, lower levels were found in the WSTF KD cells (see Figure S1C). (TIF) [file pone.0019184.s001.tif]

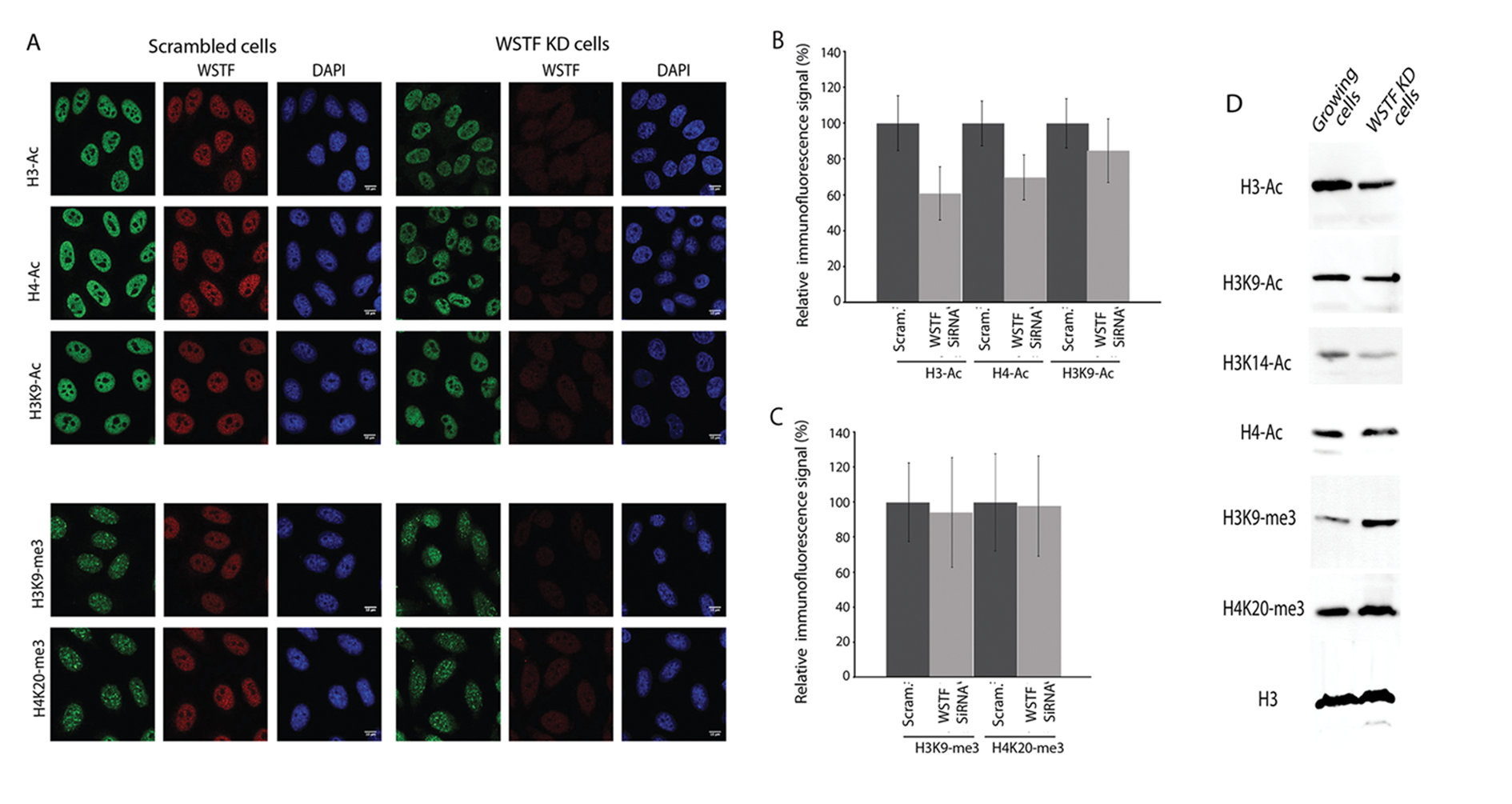

Supplement: Figure S2 — WSTF increases histone acetylation globally. (A) Immunolocalisations of WSTF and modified histones. Cells were fixed for 15 min with formaldehyde 3.7% at room temperature, permeabilised with Triton X-100 0.5% for 7 min, and blocked with 0.5% milk for 30 min. Primary antibodies H3-Ac, H4-Ac, H3K9-Ac, H3K9-me3, H4K20-me3, and WSTF were incubated for one hour, and the secondary antibodies coupled either to Cy2 or Alexa 568 (Jackson, Invitrogen) for 45 min at RT. DNA was revealed by DAPI staining (300 nM for 3 min at RT) and coverslips were mounted with Mowiol (Merck). Images were obtained with a confocal microscope (Zeiss LSM 510 meta) with 63X oil objective of NA 1.3. Localisation of histones (green) and WSTF (red) in control cells (left panels, scrambled cells) or after silencing of WSTF expression (right panels, WSTF KD cells). The chromatin is revealed by DAPI labelling (blue). Scale bar, 10 µm. The upper panel shows the localisations of acetylated forms of histones H3-Ac, H4-Ac and H3K9-Ac (upper, middle and lower row). The lower panel shows the localisations of methylated histones H3K9-me3 and H4K20-me3 (upper and lower row). (B and C) Quantification of fluorescence signals obtained after immunostaining histones, scrambled control cells and WSTF-silenced cells. Micrographs were analysed with ImageJ; the mean gray value of an individual nucleus was registered. The mean gray values were averaged and expressed as percentages. The average mean gray value of the controls was set as 100% of signal and the average of the mean gray values measured after silencing of WSTF expression was expressed proportionally. In each experiment the number of nuclei measured was between 45 and 74. Error bars represent standard deviations. (D) Immunoblots (15% SDS-PAGE) of nuclear extracts from WSTF KD cells and scrambled control cells. The antibodies used are indicated to the left. Histone H3 is used as a loading control. (TIF) [file pone.0019184.s002.tif]

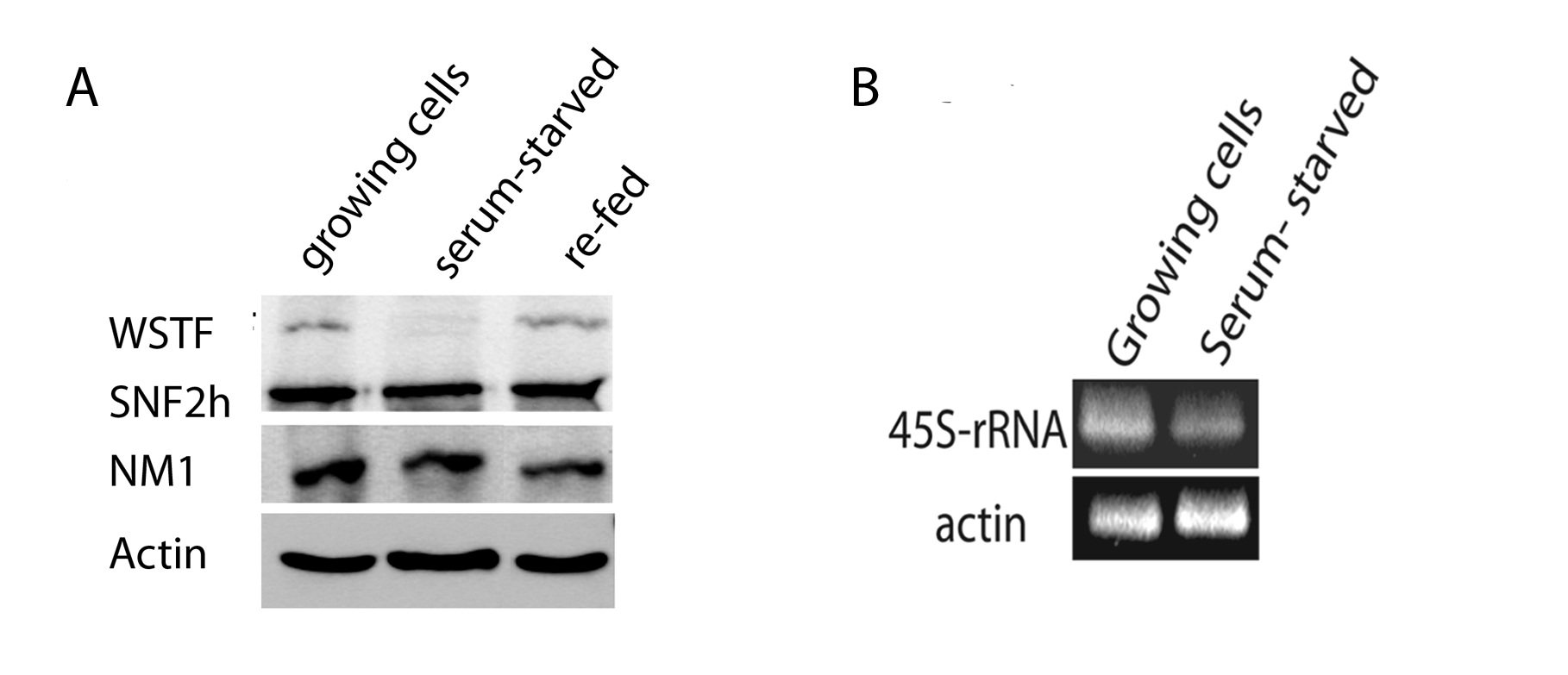

Supplement: Figure S3 — The 45S rRNA level and the WSTF protein level are reduced in serum-starved cells. (A) Immunoblot (7% SDS-PAGE) of 30 µg of cell lysates (0.7 M KCl) from growing cells, serum-starved cells and re-fed cells (9 h), using antibodies against WSTF, SNF2h, and NM1, as indicated at the left. Actin was used as a loading control. (B) 45S rRNA levels in growing cells and in serum-starved cells, detected from reverse transcriptase (Invitrogen) converted RNA preparations, and amplified with primer pair 0.3 kb (see Fig. 1C). Primer pair detecting actin was used as a control. (TIF) [file pone.0019184.s003.tif]

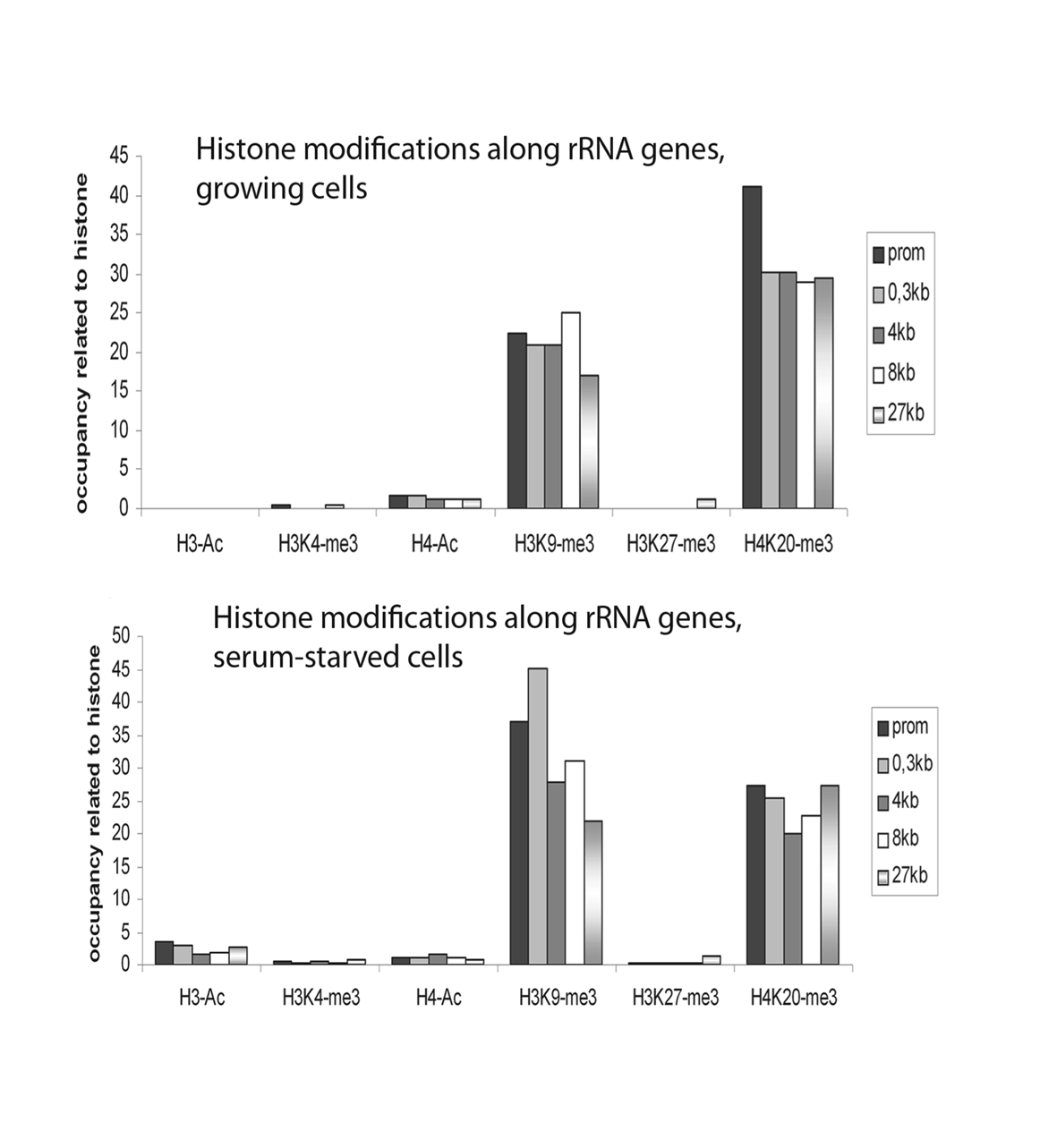

Supplement: Figure S4 — Growing cells have low levels of H3-Ac along the rRNA gene. (A) ChIP of growing cells (upper panel) and ChIP of serum-starved cells (lower panel) with antibodies indicated below, in which PCR primers at the positions along the rDNA repeat as indicated were used. One representative experiment out of six is presented with the values adjusted to the signal for the relevant histone for each primer. (TIF) [file pone.0019184.s004.tif]

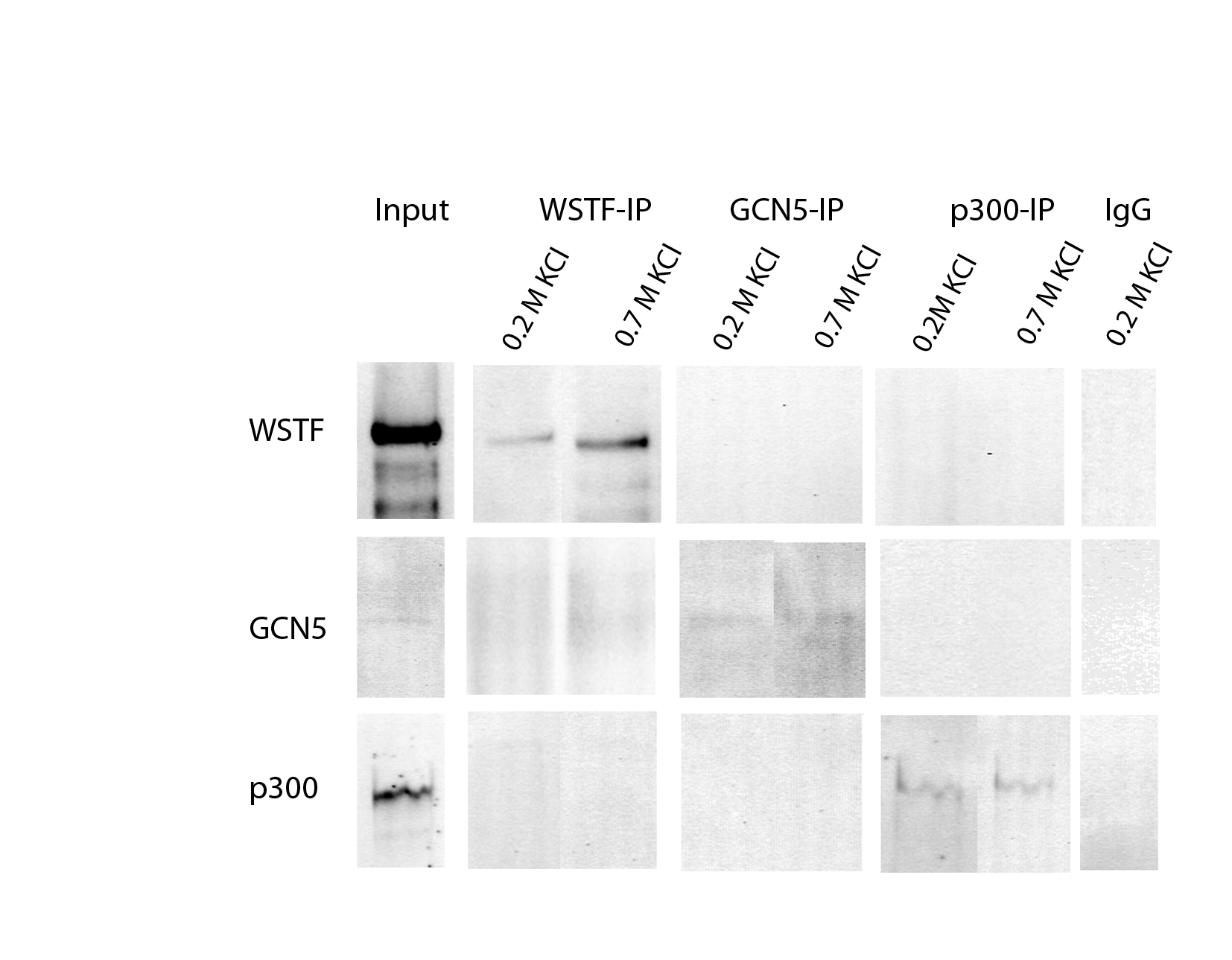

Supplement: Figure S5 — GCN5 and p300 does not interact directly with WSTF. Immunoblot (10% SDS-PAGE) of immunoprecipitations using antibodies against WSTF, GCN5 and p300, as indicated, of nuclear extracts prepared at 0.2 M KCl and 0.7 M KCl. Co-immunoprecipitated proteins were detected with the antibodies marked at the left. IgG was used as a control. (TIF) [file pone.0019184.s005.tif]
