# Supplementary material for: The Chromatin Remodelling Complex B-WICH Changes the Chromatin Structure and Recruits Histone Acetyl-Transferases to Active rRNA Genes
Source: PLoS One. 2011 Apr 29;6(4):e19184. doi: 10.1371/journal.pone.0019184 (PMC3084792; doi:10.1371/journal.pone.0019184)
Supplement: Table S1 — Primers used in the study (based on the human rRNA gene repeat, U13369). (DOC) [file pone.0019184.s006.doc]

Supporting Table 1:

| **Primers** | **Forward** | **Reverse** |
| --- | --- | --- |
| **45S [62]** | 5´- CTC CGT TAT GGT AGC GCT GC -3´ | 5´- GCG GAA CCC TCG CTT CTC -3´ |
| **promoter pair 1 [62]** | 5´- GGT ATA TCT TTC GCT CCG AG - 3´ | 5´- AGC GAC AGG TCG CCA GAG GA - 3´ |
| **Promoter pair 2 [63]** | 5´- AGC GAC AGG TCG CCA GAG GA - 3´ | 5´- GCG ATG GTG GCG TTT TTG G -3´ |
| **H1 [15]** | 5´- GGC GGT TTG AGT GAG ACG AGA - 3´ | 5´- ACG TGC GCT CAC CGA GAG CAG - 3´ |
| **H4 [15]** | 5´- CGA CGA CCC ATT CGA ACG TCT - 3´ | 5´- CTC TCC GGA ATC GAA CCC TGA - 3´ |
| **H8 [15]** | 5´- AGT CGG GTT GCT TGG GAA TGC - 3´ | 5´- CCC TTA CGG TAC TTG TTG ACT - 3´ |
| **H18 [15]** | 5´- GTT GAC GTA CAG GGT GGA CTG - 3´ | 5´- GGA AGT TGT CTT CAC GCC TGA - 3´ |
| **H27 [15]** | 5´- CCT TCC ACG AGA GTG AGA AGC G - 3´ | 5´- CTC GAC CTC CCG AAA TCG TAC - 3´ |
