# Supplementary material for: The Chromatin Remodelling Complex B-WICH Changes the Chromatin Structure and Recruits Histone Acetyl-Transferases to Active rRNA Genes
Source: PLoS One. 2011 Apr 29;6(4):e19184. doi: 10.1371/journal.pone.0019184 (PMC3084792; doi:10.1371/journal.pone.0019184)
Supplement: Table S2 — Primers used in the high resolution MNase assay, investigating 1 kb upstream of the transcription start site. (DOC) [file pone.0019184.s007.doc]

Supporting Table 2:

| **Primer pair** | **Forward** | **Reverse** |
| --- | --- | --- |
| 42012 | 5’-gcttctcgactcacggtttc | 5’-gtagaccgcgcggcgaaac |
| 42090 | 5’-gttgtcgggctccatctgg | 5’-cgacactagatccgtctcg |
| 42135 | 5’-cttccggagctgcggtgg | 5’-ctttcgggcgccagcagt |
| 42181 | 5’-gctgtgagctaggcagagc | 5’-ccagcgaacactcagtgtc |
| 42235 | 5’-ggtggcgccagagctgtg | 5’-ctccgacagcgacgcgaag |
| 42281 | 5’-cgtgcaggtttatgtggg | 5’-gcatcgagggctccgggc |
| 42370 | 5’-gtagctcccgaggcccg | 5’-ccggcttaagcaaaggctc |
| 42453 | 5’-cgctcatcctggccgtc | 5’-gagacggcgctaggaaagac |
| 42589 | 5’-gatcctttctggcgagtcc | 5’-ggcttttacgaaggccgag |
| 42760 | 5’-cgtggattccggaagagcc | 5’-ggagggacgaaggctctc |
| 42808 | 5’-gtccttgggttgaccagag | 5’-gtccacaggcacaggcacag |
| 42854 | 5’-gcgatggtggcgtttttgg | 5’-ccctccatatagaaagcgag |
| 2c | 5’-ctgacacgctgtcctctgg | 5’-ggcgcgaggacggaactc |
| 85c | 5’-ctagccggccgcgctcc | 5’-ccttccgccgctcccgg |
| 200c | 5’-gaggcggcgagggccac | 5’-cacagtccgcaagagcag |
| 300c (hr1) [59] | 5’-tgtcaggcgttctcgtctc | 5’-gagagcacgacgtcaccac |
| 1 kb c (H1) [15] | See Table S1 |  |
| 4 kb c (H4) [15] | See Table S1 |  |
|  |  |  |
